# Dietary meat mutagens intake and cancer risk: A systematic review and meta-analysis

**DOI:** 10.3389/fnut.2022.962688

**Published:** 2022-09-23

**Authors:** Qie Reng, Ling Ling Zhu, Li Feng, Yong Jie Li, Yan Xing Zhu, Ting Ting Wang, Feng Jiang

**Affiliations:** ^1^Taizhou Central Hospital (Taizhou University Hospital), Taizhou, China; ^2^Taizhou Jiaojiang Maternal and Child Health Care Hospital, Taizhou, China; ^3^Guizhou Provincial People’s Hospital, Guiyang, Guizhou, China; ^4^College of Pharmacy, Shaoyang University, Shaoyang, China; ^5^Guizhou Orthopedics Hospital, Guiyang, Guizhou, China

**Keywords:** meat mutagens, heterocyclic amines (HCAs), polycyclic aromatic amines (PAHs), cancer risk, meta-analysis

## Abstract

**Background:**

Clinical and preclinical studies suggested that certain mutagens occurring as a reaction of creatine, amino acids, and sugar during the high temperature of cooking meat are involved in the pathogenesis of human cancer. Here we conducted a systematic review and meta-analysis to examine whether meat mutagens [PhIP, MeIQx, DiMeIQx, total HCA, and B(a)P] present a risk factor for human cancer.

**Methods:**

We searched the following databases for relevant articles published from inception to 10 Oct 2021 with no language restrictions: Pubmed, Embase, Cochrane Central Register of Controlled Trials (CENTRAL), Baidu Academic, Zhejiang Digital Library. Two independent researchers screened all titles and obtained eligible texts for further screening. Independent data extraction was conducted, and meta-analysis was carried out using random-effects models to calculate the risk ratio of the meat mutagens exposure.

**Results:**

A total of 1,786,410 participants and 70,653 cancer cases were identified. Among these, there were 12 different types of cancer at various sites, i.e., breast, bladder, colorectal, colon, rectum, prostate, lung, Non-Hodgkin lymphoma, kidney, gastric, esophagus, pancreatic, hepatocellular carcinoma. Cancer risk was significantly increased by intake of PhIP (OR = 1.13;95% CI 1.07–1.21; *p* < 0.001), MeIQx (OR = 1.14; 95% CI: 1.07–1.21; *p* < 0.001), DiMeIQx (OR = 1.07; 95% CI: 1.01–1.13; *p* = 0.013), total HCA (OR = 1.20; 95% CI: 1.03–1.38; *p* = 0.016), and cancer risk was not significantly increased by intake of B(a)P (OR = 1.04; 95% CI: 0.98–1.10; *p* = 0.206).

**Conclusion:**

Meat mutagens of PhIP, MeIQx, DiMeIQx, and total HCA have a positive association with the risk of cancer.

**Systematic review registration:**

[www.crd.york.ac.uk/prospero], identifier [CRD42022148856].

## Introduction

**FIGURE 1:**
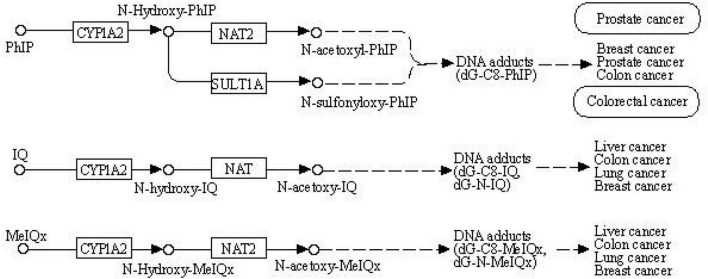
The Kegg pathway for meat mutagens to be metabolically.

The World Cancer Research Fund and American Institute for Cancer Research provided convincing evidence on the association between red meat consumption and colorectal cancer risk (1). Processed meat has become a matter of public health concern since several epidemiological studies have indicated that high meat consumption correlates with higher rates of cancer and other chronic diseases (2–6). Animal and human studies suggested that certain mutagens occurring as a reaction of amino acids and sugar during the high temperature of cooking meat are involved in the pathogenesis of human cancer, such as heterocyclic amines (HCAs) and polycyclic aromatic amines (PAHs) (7–10). HCAs and PAHs are a group of mutagenic compounds that include 2-amino-1-methyl-6-phenylimidazo (4,5-b) pyridine (PhIP), 2-amino-3,8-dimethylimidazo (4,5-f) quinoxaline (MeIQ) 2-amino-3,4,8-trimethylimidazo (4,5-f) quinoxaline (DiMeIQx) and benzo(a)pyrene (BaP) (11–13). These mutagenic compounds have been presumed to increase the occurrence of tumors (2, 14–17). The mechanism is similar to other environmental chemical carcinogens, and metabolism enzymes metabolically activate meat mutagens (18–20). In the first step, cytochrome P450 (CYP) enables HCAs and PAHs to activate and form genotoxic electrophilic intermediates (21). In the second step, activated metabolites are detoxified by *N*-acetyltransferase 2 (NAT2) with *N*-acetylation and *O*-acetylation (22). This process is shown in the Kegg pathway diagram in Figure 1.

Meta-analysis can take into account a large amount of evidence (i.e., research) on a topic, which is desirable to identify a clear relationship between the variables of concern. In this study, we study the risk estimate about meat mutagens and human cancer. We conducted a systematic review and meta-analysis to shed light on the relation between meat mutagens (PhIP, MeIQx, DiMeIQx, and BaP) and different types of human cancers. The study have registered on PROSPERO. ID: CRD42022148856.

Our objectives were as follows:

(i) Conduct a meta-analysis to examine whether meat mutagens [PhIP, MeIQx, DiMeIQx, total HCA, and B(a)P] present a risk factor for human cancer.

(ii) To identify which types of human cancers are especially vulnerable to meat related mutagens.

(iii) To identify which categories of meat mutagens that warrant further in-depth evaluation according to harmfulness.

## Methods

In this study, meta-analysis was according to PRISMA (Preferred Reporting Items for Systematic reviews and Meta-Analyses).

### Search strategy

Exposure referred to heterocyclic amines (PhIP, MeIQx, DiMeIQx, total HCA) and benzo (a)pyrene; the outcomes of interest included all kinds of cancer. Following databases were searched from inception to 10 Oct 2021 with no language restrictions: PubMed, Cochrane Central Register of Controlled Trials (CENTRAL), Baidu Schoolar, Google scholar, and Zhejiang Digital Library. We used the following search keywords [meat mutagens OR heterocyclic amin OR PhIP OR MeIQx OR DiMeIQx OR benzo(a)pyrene] and (neoplasm OR cancer OR tumor). To maximize the search for relevant articles, the reference lists of identified articles and relevant systematic reviews were examined to identify additional relevant publications.

### Inclusion criteria

Articles were considered eligible if they met the following inclusion criteria: (i) study design: epidemiological study and clinical study with case-control or cohort study; (ii) object: the association between heterocyclic amine [PhIP, MeIQx, DiMeIQx, benzo(a)pyrene] intake and cancer risk; (iii) outcomes: publications presented results of relative risk [RR], odds ratio [OR], or hazard ratio (HR) for the highest vs. lowest exposure.

If more articles were produced based on the same cohort study, such as Nurses Health Study (NHS), and analyses presented utilized unique sites or exposures, the article with the largest number of cases was selected for statistical analysis. No studies were excluded due to issues related to data quality or design.

### Exclusion criteria

(i) Study design with cross-sectional surveys, ecologic analyses, case reports, editorials.

(ii) Exposure comes from the working environment or smoking.

(iii) Experimental study on the animal model.

### Study selection and data extraction

After the removal of duplicates, two independent researchers screened all titles and obtained eligible texts for further screening. Disagreements between researchers were solved by the third author.

The following data were extracted from the included studies; (i) basic information: first author, year of publication, study design, study region; (ii) individual data: sample size, tumor site, dietary assessment method, exposure quantification method, risk estimates [rate ratios (RR); odds ratio (OR); hazard ratio (HR); corresponding 95% confidence interval (CI)], (iii) matched or adjusted variables. We abstracted those that adjusted for multivariate or most confounding factors.

### Statistical analysis

The random-effect model of the inverse variance weighting test was used to calculate OR and the corresponding 95% CI for the highest vs. the lowest level of exposures. *P* < 0.05 was considered statistically significant; *p*-values and *I*^2^ values were used for the heterogeneity test.

### Subgroup analyses

We performed subgroup analyses according to cancer site, study design, and country. Forest plots were generated for PhIP, MeIQx, DiMeIQx, total HCA, and B(a)P. Subgroup analysis can be used to evaluate the potential association between exposure and influencing factors and describe sources of heterogeneity by stratifying factors.

### Publication bias and sensitivity analyses

The existence of publication bias was evaluated by Begg’s test and Egger’s test, the Begg methods test was also used for funnel plot asymmetry. If the value of Begg’s Test (Pr > | z|) and Egger’s test Pr > | z| were < 0.05, the funnel plot was considered to be asymmetrical, thus suggesting the potential existence of publication bias. When potential bias was detected, a trim-and-fill analysis was further performed to assess the influence of the bias and to have the bias-corrected. We also performed a sensitivity analysis to investigate the influence of a single study on the pooled OR estimate by omitting one study in each turn. Stata12.0 software was used for all analyses.

## Results

Among 1,141 publications that were initially identified from the data sources, 58 were included in the final meta-analysis. Details of the selection of publications included in the meta-analysis are shown in Supplementary Figure 1. A total of 1,786,410 participants, 70,653 cancer cases, and 12 types of cancer at various sites, i.e., breast, bladder, colorectal, colon, rectum, prostate, lung, Non-Hodgkin lymphoma, kidney, gastric, esophagus, pancreatic, and hepatocellular carcinoma were investigated. All studies used a food frequency questionnaire (FFQ) to collect dietary information, and most of the publications used the online Computerized Heterocyclic Amines Resource for Research to estimate heterocyclic amines intake from the Epidemiology of Disease (CHARRED) database.

Table 1 shows the main characteristics and results of the 58 publications in this meta-analysis. As several publications reported the risk for more than one cancer site, the total number of trials included in this meta-analysis amounted to 67. When some large cohort studies had been researched more than one time with different cancer sites, only one publication had been selected to statistics the number of cases to avoid repeating counting.

**TABLE 1:** Characteristics of studies included in the meta-analysis.

Cancer site; first author	Country	Year	Study design	Cases	High vs. low level of intake RR (95% CI)				
					
					PhIP	MeIQx	DiMeIQx	Total HCAs	B(a)P
**Breast**									
Eduardo D.S (29)	Uruguay	1997	Case-Control	352/382	2.59(1.42–4.70)	2.31(1.27–3.55)			
Rashmi Sinha (32)	USA	2000	Case-Control	273/657	1.9(1.1–3.4)	1.0(0.5–2.1)	0.80(0.4–1.5)		
Ralaph J D (33)	USA	2000	Case-Control	114/280	0.38(0.17–0.86)	0.55(0.26–1.19)	0.50(0.22–1.15)		
Susan E. Steck (34)	USA	2007	Case-Control	1,508/1,556	0.92(0.70–1.22)	0.94(0.71–1.25)	0.91(0.66–1.26)		1.01(0.68–1.50)
LM Ferrucci (35)	USA	2009	Cohort	1,205/52,158	1.11(0.92–1.34)	1.26(1.03–1.55)	1.18(0.98–1.42)		1.01(0.83–1.23)
Laura I. Mignone (36)	USA	2009	Case-Control	2,686/3,508	0.96(0.81–1.13)	0.94(0.80–1.10)	0.94(0.81–1.11)		
Geoffrey C. Kabat (37)	USA	2009	Cohort	3,818/120,755	0.98(0.88–1.09)	1.00(0.89–1.11)	0.95(0.86–1.04)		0.96(0.88–1.06)
Kana Wu (38)	USA	2011	Cohort	2,317/533,618	0.92(0.80–1.05)	0.90(0.79–1.03)	0.92(0.80–1.05)	0.98(0.85–1.12)	
**Bladder**									
Katarina Augustsson (30)	Sweden	1999	Case-Control	273/553	1.2(0.7–2.1)	1.1(0.6–1.9)	1.00(0.60–1.7)		
Reina Garcia Closas (39)	Spanish	2007	Case-Control	912/873	1.2(0.8–1.7)	1.2(0.8–1.6)	1.30(0.9–1.8)		
Paula Jakszyn (40)	European	2011	Cohort	1,001/481,419					
Leah M. Ferrucci (41)	USA	2011	Cohort	854/300,933	1.19(0.95–1.48)	0.93(0.75–1.15)	1.08(0.90–1.30)		0.95(0.77–1.17)
Jie Lin (42)	USA	2012	Case-Control	884/878	2.67(1.2–5.96)	6.07(2.24–16.4)	3.44(1.5–7.89)	3.32(1.37–8.01)	2.03(0.9–4.58)
Colorectal*									
Katarina Augustsson (Colon) (30)	Sweden	1999	Case-Control	352/553	0.6(0.4–0.9)	0.6(0.4–1.0)	0.6(0.4–0.9)		
Katarina Augustsson (Rectum) (30)	Sweden	1999	Case-Control	352/553	0.6(0.4–1.1)	0.7(0.4–1.2)	0.6(0.4–1.1)		
Loic Le M (Colon) (43)	USA	2002	Case-Control	727/727	1.0(0.6–1.6)	1.0(0.6–1.1)	1.10(0.70–1.7)	1.0(0.6–1.6)	
Loic Le Marchand (Rectum) (43)	USA	2002	Case-Control	727/727	1.7(0.3–3.8)	3.1(1.3–7.7)	2.70(1.10–6.3)	2.20(1.0–4.7)	
Susan Nowell (44)	USA	2002	Case-Control	156/366		4.09(1.94–9.08)			
L.M.Butler (Colon) (45)	USA	2003	Case-Control	620/1,038	0.9(0.6–1.5)	1.1(0.6–2.0)	1.8(1.1–3.1)		1.20(0.80–1.7)
Ute Nöthlings (46)	USA	2009	Case-Control	389/1,444	1.03(0.77–1.39)	1.09(0.81–1.47)	1.18(0.88–1.59)	1.03(0.77–1.39)	
MINATSU KOBAYASHI (47)	Japan	2009	Case-Control	117/238	1.32(0.27–6.48)	1.23 (0.23–6.64)	1.98(0.42–9.32)	0.99(0.21–4.81)	
Amanda J. Cross (48)	USA	2010	Cohort	2,719/300,948	0.99(0.87–1.12)	1.19(1.05–1.34)	1.17(1.05–1.29)		0.96(0.85–1.08)
Hansong Wang (49)	USA	2010	Case-Control	498/609	1.20(0.86–1.68)	1.10(0.78–1.54)	0.99(0.71–1.37)	1.07(0.76, 1.51)	
Nicholas J.O (50)	USA	2012	Cohort	3,404/131,763	0.95(0.81–1.11)	1.01(0.86–1.19)	0.88(0.75–1.03)	0.90(0.76–1.05)	
Drew S. Helmus (colon) (51)	USA	2013	Case-Control	1,062/1,645	1.18(0.91–1.52)	1.87(1.44–2.44)	1.67(1.29–2.17)		0.87(0.68–1.12)
Paige E. Miller (52)	USA	2013	Case-Control	989/1,033	1.06(0.79–1.43)	1.22(0.91–1.64)	1.48(1.12–1.96)		0.90(0.67–1.21)
Ngoan Tran Le (53)	USA	2016	Cohort	418/29,615	1.01(0.72–1.41)	1.22(0.89–1.68)	0.88(0.65–1.19)		
Sanjeev Budhathoki (54)	Japan	2019	Case-Control	302/403	0.76(0.48–1.20)	0.90(0.57–1.43)	0.84(0.53–1.34)	0.84(0.53–1.34)	
Seyed Mehdi Tabatabaei (16)	Australia	2010	Case-Control	575/709					0.96(0.67–1.36)
Hang Viet Dao (55)	Viet Nam	2020	Case-Control	512/1,096	4.89(3.03–7.89)	Hang Viet Dao	Viet Nam	2020	Case-Control
**Prostate**									
Alan E.Norrish (17)	New Zealand	1999	Case-Control	317/480	1.05(0.70–1.59)	0.97(0.63–1.49)	1.24(0.82–1.87)	1.09(0.72–1.65)	
Amanda J. Cross (56)	USA	2005	Cohort	1,338/29,361	1.06(0.78–1.43)	0.95(0.64–1.43)	1.03(0.69–1.53)		0.85(0.64–1.13)
Stella Koutros (57)	USA	2008	Cohort	668/197,017	1.04(0.82–1.32)	1.15(0.90–1.47)	1.19(0.93–1.51)		0.91(0.71–1.16)
Stella Koutros (58)	USA	2009	Case-Control	1,230/1,204	1.11(0.86–1.44)	0.80(0.57–1.12)	0.91(0.65–1.27)		
Rashmi Sinha (59)	USA	2009	Cohort	10,313/175,343	1.00(0.92–1.09)	0.98(0.90–1.08)	1.00(0.93–1.08)		1.09(1.00–1.18)
Sander.A (60)	Germany	2010	Cohort	377/9,578	0.89(0.66–1.22)	1.06(0.77–1.45)	0.98(0.72–1.34)		
Sanoj Punnen (61)	USA	2011	Case-Control	470/512	1.32(0.86–2.05)	1.69(1.08–2.64)	1.53(1.00–2.35)		1.34(0.87–2.07)
Amit D.Joshi (62)	USA	2012	Case-Control	1,857/1,096	1.2(0.9–1.6)	1.0(0.8–1.4)	1.0(0.8–1.3)		0.90(0.70–1.1)
Esther M. John (63)	USA	2012	Case-Control	726/527	1.05(0.73–1.53)	0.93(0.64–1.35)	0.88(0.61–1.25)		1.02(0.72–1.47)
Jacqueline M Major (31)	USA	2012	Cohort	1,089/7,949	1.03(0.84–1.26)	1.12(0.90–1.38)	1.30(1.05–1.61)		0.94(0.76–1.16)
Sabine Rohrmann (64)	USA	2016	Cohort	2,770/26,030	1.08(0.95–1.22)	1.12(0.98–1.27)	1.09(0.97–1.21)	1.13(1.00–1.28)	
Masahide Koda (20)	Japan	2017	Case-Control	750/870	1.84(1.35–2.50)	2.25(1.65–3.06)		1.90(1.40–2.59)	
**lung**									
Rashmi Sinha (65)	USA	2000	Case-Control	593/623	0.9(0.8–1.1)	1.5(1.1–2.0)	1.2(0.9–1.6)		
Tram Kim Lam (66)	USA	2009	Case-Control	2,101/2,120	1.5(1.2–1.8)	1.4(1.2–1.7)	1.0(0.8–1.2)		1.30(1.1–1.6)
Paolo Boffetta (67)	Uruguay	2009	Case-Control	846/846	2.16(1.48–3.15)	1.96(1.35–2.85)			2.08(1.43–3.01)
NatasaTasevska (68)	USA	2009	Cohort	2,279/278,380	1.11(0.97–1.27)	1.20(1.04–1.38)	1.06(0.94–1.19)		1.09(0.95–1.24)
NatasaTasevska (68)	USA	2009	Cohort	1,327/189,596	1.03(0.86–1.23)	0.95(0.80–1.13)	0.91(0.78–1.06)		0.96(0.81–1.13)
**non-Hodgkin lymphoma**									
Amanda J.Cross (69)	USA	2005	Case-Control	458/383	0.73(0.46–1.16)	1.01(0.64–1.61)	0.63(0.41–0.97)		0.73(0.46–1.14)
Carrie R. Daniel (70)	USA	2012	Cohort	3,611/302,162	0.98(0.86–1.12)	0.90(0.78–1.04)	1.01(0.90–1.14)		1.04(0.9–1.19)
**Kidney**									
E De Stefanil (71)	Uruguay	1998	Case-Control	121/234	2.18(1.14–4.19)				
Katarina Augustsson (30)	Sweden	1999	Case-Control	352/553	0.9(0.5–1.7)	0.9(0.5–1.9)	1.10(0.60–2.0)		
CR Daniel (72)	USA	2011	Case-Control	1,192/1,175	0.92(0.68–1.26)	1.11(0.80–1.55)	0.94(0.69–1.27)		1.11(0.87–1.42)
Carrie R Daniel (73)	USA	2012	Cohort	1,814/492,186	1.30(1.07–1.58)	1.16(0.96–1.42)	0.96(0.81–1.12)		1.23(1.01–1.48)
**Gastric**									
Eduardo De Stefani (74)	Uruguay	1998	Case-Control	340/698	3.86(2.34–6.37)				
Paul D. Terry (75)	Sweden	2003	Case-Control	258/815	1.02(0.8–1.9)	1.30(0.80–2.0)	1.20(0.80–1.9)	1.30(0.80–2.1)	
MINATSU KOBAYASHI (76)	Japan	2009	Case-Control	149/396	1.33(0.44–4.02)	1.06(0.36–3.12)	0.81(0.36–1.82)	1.11(0.36–3.49)	
Amanda J. Cross (77)	USA	2011	Cohort	501/337,074	1.22(0.82–1.83)	0.83(0.57–1.22)	0.97(0.68–1.39)		0.99(0.67–1.46)
**esophagus**									
Eduardo De Stefani (78)	Uruguay	1998	Case-Control	140/286	2.50(1.20–5.20)	1.20(0.60–2.50)	1.40(0.6–2.7)	2.30(1.10–5.0)	
Paul D. Terry (75)	Sweden	2003	Case-Control	165/815	1.5 (0.9–2.7)	1.7 (1.0–2.8)	1.60 (1.0–2.8)	2.40(1.20–4.8)	
Amanda J. Cross (77)	USA	2011	Cohort	215/337,074	1.09(0.60–1.97)	0.96(0.53–1.75)	1.00(0.58–1.73)		0.70(0.39–1.26)
**Pancreatic**									
Kristin E. Anderson (79)	USA	2005	Case-Control	193/674	1.80(1.00–3.10)	1.50(0.90–2.70)	2.00(1.20–3.50)		2.20(1.20–4.0)
Donghui Li (80)	USA	2007	Case-Control	626/530	1.30(0.87–1.94)	1.11(0.75–1.65)	1.52(1.03–2.25)		1.32(0.89–1.97)
Rachael Z. S (81)	USA	2007	Cohort	836/332,913	1.17(0.88–1.56)	1.22(0.91–1.64)	1.29(1.01–1.64)		1.01(0.76–1.34)
Kristin E. Anderson (82)	USA	2012	Cohort	248/62,581	1.15(0.76–1.74)	1.75(1.11–2.76)	1.81(1.20–2.74)		0.97(0.62–1.52)
**Hepatocellular carcinoma**									
Yanan Ma (83)	USA	2019	Cohort	163/121,700	1.24(0.73–2.08)	1.30(0.78–2.1)	1.05(0.62–1.7)		

*Several publications reported colorectal cancer, while others reported by colon cancer or rectum cancer separately.

### PhIP

**FIGURE 2:**
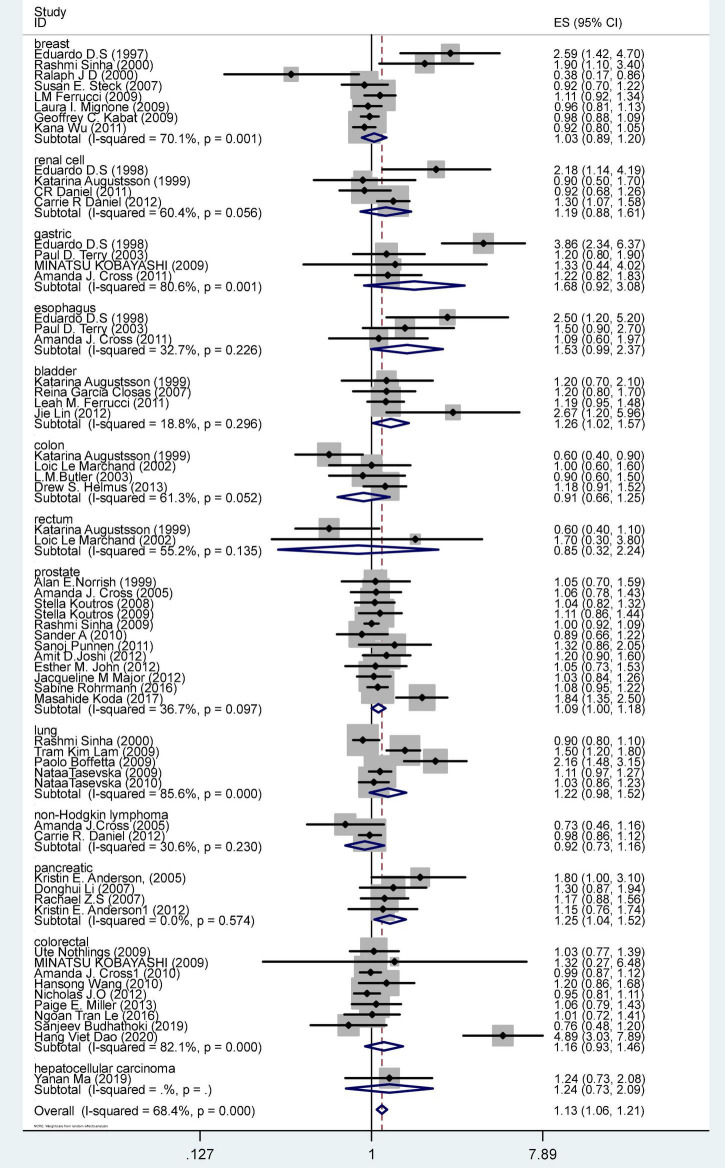
Forest plot of PhIP intake and cancer stratified by cancer site.

Figure 2 shows the OR and 95% CI for PhIP intake and cancer risk. The OR was 1.13 (CI 1.07–1.21), *p* = < 0.001. The result of the meta-analysis was a significant summary risk estimate.

### MeIQx

Supplementary Figure 6 displays the pooled ORs and 95% CI for the highest vs. the lowest level of MeIQx intake according to subgroups of the cancer site. The OR was 1.14 (CI 1.07–1.21, *p* = 0.000). The meta-analysis revealed a significant association between MeIQx intake and carcinoma risk.

### DiMeIQx

Supplementary Figure 8 displays the pooled ORs and 95% CI for the highest vs. the lowest level of DiMeIQx intake according to subgroups of the cancer site. The OR was 1.07 (CI 1.01–1.13, *p* = 0.013). The results indicated a weakly significant association between DiMeIQx intake and carcinoma risk.

### Total heterocyclic amines

Sixteen publications evaluated the association between total HCA consumption and cancer (Supplementary Figure 10). The OR was 1.20 (CI 1.03–1.38, *p* = 0.016). The results revealed a statistically significant association between total HCA intake and carcinoma risk.

### B(a)P

Thirty studies were included in the meta-analysis of the association between B(a)P intake and cancer risk (Supplementary Figure 12). The OR was 1.04 (CI 0.98–1.10), *p* = 0.206. The results revealed no statistically significant association between B(a)P intake and carcinoma risk.

### Subgroups analysis

Table 2 shows the summary OR and 95% CI for high vs. low levels of PhIP, MeIQx, DiMeIQx, total HCAs, and B(a)P intake and cancer risk according to subgroups of the cancer site. First, no risk emerged for breast cancer, non-Hodgkin lymphoma, and gastric cancer. For bladder cancer, OR was 1.26 (CI 1.02–1.57) for PhIP, and 3.32 (CI 1.37–8.03) for total HCA, respectively. As for colorectal cancer, OR was 1.16 (CI 1.01–1.33) for MeIQx, and in rectum cancer, it was 2.20 (CI 1.01–4.77) for total HCAs, respectively. Concerning prostate cancer, a borderline significantly increased OR of 1.09 (CI 1.00–1.18) was found for PhIP. Lung cancer had significantly increased OR = 1.31 (CI 1.07–1.60) for MeIQx. Kidney cancer had significantly increased OR = 1.18 (CI 1.02–1.38) for B(a)P; esophagus cancer had OR = 2.35 (CI 1.4–3.93) for total HCA, while in pancreatic cancer, OR was 1.25 (CI 1.04–1.52) for PhIP, 1.31 (CI 1.08–1.59) for MeIQx, 1.50 (CI 1.24–1.82) for DiMeIQx, respectively.

**TABLE 2:** Results of meta-analyses of epidemiological studies of Meat Mutagens intake and cancer risk.

Cancer site	Cases	High vs. low level of intake OR (95% CI)									
		
		NO	PhIP	NO	MeIQx	NO	DiMeIQx	NO	Total HCA	NO	B(a)P
Breast	12,273/712,914	8	1.03(0.89–1.20)	8	1.03(0.89–1.18)	7	0.96(0.88–1.04)	1	0.98(0.85–1.13)	3	0.97(0.89–1.05)
Bladder	3,924/784,656	4	1.26(1.02–1.57)	4	1.35(0.85–2.14)	4	1.28(0.93–1.77)	1	3.32(1.37–8.03)	2	1.25(0.61–2.55)
Colorectal	13,919/48,134	9	1.16(0.93–1.46)	9	1.16(1.01–1.33)	7	1.06(0.91–1.23)	5	0.94(0.83–1.07)	3	0.95(0.86–1.06)
Colon	2,761/3,963	4	0.91(0.66–1.25)	4	1.07(0.65–1.77)	4	1.19(0.72–1.96)	1	1.00(0.61–1.63)	2	0.99(0.73–1.35)
Rectum	1,079/1,280	2	0.85(0.32–2.24)	2	1.41(0.33–6.05)	2	1.22(0.28–5.13)	1	2.20(1.01–4.77)		
Prostate	21,905/449,967	12	1.09(1.00–1.18)	12	1.11(0.98–1.26)	11	1.07(0.99–1.15)	3	1.32(0.94–1.87)	7	1.00(0.91–1.10)
Lung	7,246/471,565	5	1.22(0.98–1.52)	5	1.31(1.07–1.60)	4	1.02(0.92–1.12)			4	1.23(0.98–1.54)
Non-Hodgkin lymphoma	4,069/302,545	2	0.92(0.73–1.16)	2	0.91(0.79–1.04)	2	0.84(0.53–1.32)			2	0.93(0.68–1.28)
Kidney	3,479/494,148	4	1.19(0.88–1.61)	3	1.13(0.96–1.33)	3	0.96(0.84–1.11)			2	1.18(1.02–1.38)
Gastric	1,252/338,893	4	1.68(0.92–3.08)	3	0.98(0.72–1.33)	2	1.06(0.80–1.39)	2	1.27(0.81–1.98)	1	0.99(0.67–1.46)
Esophagus	520/338,175	3	1.53(0.99–2.37)	3	1.28(0.92–1.84)	3	1.24(0.92–1.68)	2	2.35(1.4–3.93)	1	0.70(0.39–1.26)
Pancreatic	1,903/396,698	4	1.25(1.04–1.52)	4	1.31(1.08–1.59)	4	1.50(1.24–1.82)			4	1.22(0.90–1.64)
Hepatocellular carcinoma	163	1	1.24(0.74–2.09)	1	1.30(0.78–2.17)	1	1.05(0.62–1.76)				
Overall	70,653/1,786,401	62	1.13(1.07–1.21)	60	1.14(1.07–1.21)	54	1.07(1.01–1.13)	16	1.20(1.03–1.38)	31	1.04(0.98–1.10)

Table 3 shows the OR estimates according to subgroups of geographic location. Nine countries were included in geographic location subgroup analyses; for America, there was a significant association between PhIP, MeIQx, DiMeIQx intake, and cancers risk; for Uruguay, a significant association was found between PhIP, total HCAs intake, and cancers risk; for Vietnam, we found a significant association between PhIP intake and cancers risk. No significant associations were observed for Sweden, Spain, Germany, Japan, New Zealand, and Australia (Supplementary Figures 2, 7, 9, 11, 13).

**TABLE 3:** Meta-analysis of high vs. low meat mutagens intake in relation to the risk of cancer, in subgroups of study location.

	No	PhIP	No	MeIQx	No	DiMeIQx	No	Total HCA	No	B(a)P
USA	44	1.07(1.02–1.13)	45	1.13(1.06–1.20)	43	1.08(1.02–1.14)	8	1.06(0.93–1.22)	30	1.04(0.98–1.10)
Uruguay	4	2.87(2.12–3.88)	2	1.75(0.93–3.30)	1	1.40(0.66–2.97)	1	2.30(1.08–4.90)		
Sweden	6	0.93(0.67–1.28)	6	0.99(0.71–1.38)	6	0.94(0.68–1.31)	2	1.68(0.93–3.03)		
Spanish	1	1.20(0.82–1.75)	1	1.20(0.85–1.70)	1	1.30(0.92–1.84)				
Germany	1	0.89(0.66–1.21)	1	1.06(0.77–1.46)	1	0.98(0.72–1.34)				
Japan	4	1.24(0.69–2.25)	4	1.32(0.69–2.51)	1	0.84(0.53–1.34)	4	1.23(0.71–2.16)		
New Zealand	1	1.05(0.70–1.58)	1	0.97(0.63–1.49)	1	1.24(0.82–1.87)	1	1.09 (0.72–1.65)		
Australia									1	0.96(0.67–1.38)
Viet Nam	1	4.89 (3.03, 7.89)								

### Publication bias and sensitivity analysis

Publication bias was detected in estimate of PhIP intake and cancer risk with Begg’s Test Pr > | z| = 0.013 and Egger’s test P > | t| = 0.003, the funnel plot appeared to be relatively asymmetric (Supplementary Figure 3). Trim and fill method were used to impute fourteen studies to the left of the mean; OR was 1.119 (CI: 1.045–1.198, *p* = 0.001), this result was the same as the original OR result, which was 1.13 (CI 1.07–1.21, *p* = 0.000) (Supplementary Figure 4).

Sensitivity analyses were used to investigate the influence of a single study on the pooled OR estimates by omitting one study in each turn. The obtained results suggested that the estimates of PhIP, MeIQx, DiMeIQx, total HCA, B(a)P were not substantially modified by any single study (Supplementary Figure 5).

## Discussion

In this meta-analysis, we investigated the relationship between the meat mutagens [PhIP, MeIQx, DiMeIQx, benzo(a)pyrene] and all typical types of cancers.

First, our results indicated a significant association between (PhIP, MeIQx, DiMeIQx, total HCA) and total cancer risk, and no association between benzo(a)pyrene and cancer risk. This research included 1,987,798 participants, 69,874 cancer cases, and 12 types of cancer at the following sites: breast, bladder, colorectal, colon, rectum, prostate, lung, Non-Hodgkin lymphoma, kidney, gastric, esophagus, and pancreas. The result clearly showed which types of cancers are particularly vulnerable to related mutagens. Future studies are needed to identify categories of meat mutagens that warrant further in-depth evaluation according to harmfulness.

Second, we conducted meta-analyses for cancer risk and meat mutagens intake stratified by geographic location. We observe an increased risk in North America with exposure to PhIP, MeIQx, DiMeIQx; in South America, with PhIP and total HCA exposure, while no increased risk was observed in Europe, Asia, and Oceania. This difference may be related to dietary structure. As is well-known, the Mediterranean diet is preferred in Europe, and a previous meta-analysis suggested that the Mediterranean diet provides significant protection from the incidence of cancer of all types (23, 24). This protective effect is mainly due to the high consumption of olive oil and tomatoes, which have antioxidant effects on cancer cells (25). Moreover, in Japan, rice and vegetables are the mainstay of the country’s diet (26). Dietary patterns may have a dominant role, not just in the quantity of meat consumption, but also for the food factor activity as a protective factor against meat mutagens intake on risk of cancer (27, 28). More cohort data is necessary regarding meat mutagens intake and protective factors of cancer risk.

### Strengths and weaknesses of this study

This systematic review and meta-analysis have several strengths. First, the present study had a large sample size and a large number of epidemiologic studies, with 63 trials being included in this meta-analysis. Second, to the best of our knowledge, this systematic review is the first that provided a comprehensive guide to the risk estimates for 12 types of cancers and meat mutagens exposure.

Furthermore, the food frequency questionnaire was used to ascertain dietary information in this meta-analysis. It is challenging to ascertain respondents’ usual exposure from an FFQ. Some early epidemiological studies included a few items of cooking methods (such as pan-frying, baking, grilling/barbequing) as surrogate measures in the food frequency questionnaire. Meat intake mutagens were calculated as follows: frequency of consumption of pan-frying meat × [(portion size) × (PhIP content for each pan-frying meat according to literature data)] (29), which was inadequate for a comprehensive assessment of meat mutagens intake. After that, some studies used color photographs to reflect the range of cooking levels for cooked meat ranging from rare to very well-done and to standardize the assessment of the preferred level of doneness in dietary surveys (30). Recently, studies attempted to use the NCI CHARRED database to estimate the amount of HCA consumption (31). Therefore, we performed subgroup analyses to investigate the preferred method for calculating meat mutagens levels in diets. We stratified the trials with the use of CHARRED and without the CHARRED database (Supplementary Figure 14); the results obtained from CHARRED database revealed slight heterogeneity (*I*^2^ = 25.8%), while the results obtained without the CHARRED database revealed large heterogeneity (*I*^2^ = 80%). Accordingly, we found that the use of the CHARRED database could effectively improve the heterogeneity from the nutritional epidemiology study.

The present study also has some limitations: heterogeneity was statistically significant in case-control studies, while it was small in a cohort study (Table 4), which suggested that large heterogeneity from case-control studies contributed to the overall heterogeneity. This might be because it is difficult for cancer patients in case-control trials to retrospect their diet. It is a commonplace defect in nutritional epidemiology, and the large time span and regional span of trials may aggravate the heterogeneity. Future studies with more detailed quantitative intake may enhance the power of evidence from case-control trials.

**TABLE 4:** Test(s) of heterogeneity of meta-analysis in subgroups of study design.

	PhIP	MeIQx	DiMeIQx	B(a)P
Case-control	*I*^2^ = 76.0%	*I*^2^ = 68.9%	*I*^2^ = 61.2%	*I*^2^ = 60.7%
Cohort	*I*^2^ = 0.00%	*I*^2^ = 44.4%	*I*^2^ = 47.8%	*I*^2^ = 2.3%
Overall	*I*^2^ = 68.4%	*I*^2^ = 65.4%	*I*^2^ = 56.7%	*I*^2^ = 45.0%

Heterogeneity calculated by formula Q = SIGMA_i{(1/variance_i)*(effect_i − effect_pooled)^2} where variance_i = [(upper limit − lower limit)/(2*z)]^2.

## Conclusion

The results indicated that PhIP, MeIQx, DiMeIQx, and total HCA have a positive effect on total cancer risk, while benzo(a)pyrene was not associated with an increased risk of cancer. Results support this basic tenet of prevention in public health, restricting processed meat intake is a healthy lifestyle. This meta-analysis paves the way for a prospective epidemiological study in meat intake and cancer risk.

## Data availability statement

The original contributions presented in this study are included in the article/Supplementary material, further inquiries can be directed to the corresponding author.

## Author contributions

FJ conducted the analysis, generated the figures, and wrote the manuscript. All authors designed and conducted the systematic review, contributed to edits and revisions of the manuscript, and approved the submitted version.
