# Supplementary material for: Dietary meat mutagens intake and cancer risk: A systematic review and meta-analysis
Source: Front Nutr. 2022 Sep 23;9:962688. doi: 10.3389/fnut.2022.962688 (PMC9537819; doi:10.3389/fnut.2022.962688)
Supplement: Supplementary file 1 [file Data_Sheet_1.docx]

**APPENDIX**

**sfigure 1: low chart of the selection of publications included in the meta-analysis 2**

**sfigure 2 : Forest plot of PHIP intake and cancer stratified by geographic location 3**

**sfigure 3: Funnel plot of PHIP intake and cancer for publication bias 4**

**sfigure 4: Funnel plot of PHIP intake and cancer with trim and fill method 5**

**sfigure 5: Sensitivity analyses of PHIP intake and cancer 6**

**sfigure 6: Forest plot of MeIQx intake and cancer stratified by cancer site 7**

**sfigure 7: Forest plot of MeIQx intake and cancer stratified by location 8**

**sfigure 8: Forest plot of DiMeIQx intake and cancer stratified by cancer site 9**

**sfigure 9: Forest plot of DiMeIQx intake and cancer stratified by location 10**

**sfigure 10: Forest plot of total HCA intake and cancer stratified by cancer site 11**

**sfigure 11: Forest plot of Total HCA intake and cancer stratified by location 12**

**sfigure 12: Forest plot of B(a)P intake and risk of cancer stratified by cancer site 13**

**sfigure 13 : Forest plot of B(a)P intake and risk of cancer stratified by location 14**

**sfigure 14: Forest plot of PHIP intake and cancer stratified by use CHARRED database 15**


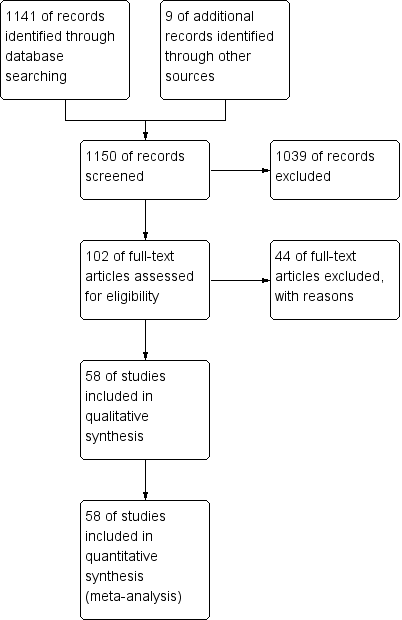


**Sfigure 1. Flow chart of the selection of publications included in the meta-analysis**

Sfigure2 : Forest plot of PHIP intake and cancer stratified by geographic location

**Phip Tests for Publication Bias**

Begg's Test

adj. Kendall's Score (P-Q) = 408

Std. Dev. of Score = 164.63 (corrected for ties)

Number of Studies = 62

z = 2.48

Pr > |z| = 0.013

z = 2.47 (continuity corrected)

Pr > |z| = 0.013 (continuity corrected)

Egger's test

------------------------------------------------------------------------------

Std_Eff | Coef. Std. Err. t P>|t| [95% Conf. Interval]

-------------+----------------------------------------------------------------

slope | -.0571551 .0476978 -1.20 0.236 -.152565 .0382547

bias | 1.199781 .390617 3.07 0.003 .4184302 1.981131

------------------------------------------------------------------------------

sfigure 3: Funnel plot of PHIP intake and total cancer

Trimming estimator: Run

Meta-analysis type: Fixed-effects model

iteration | estimate Tn # to trim diff

----------+--------------------------------------

1 | 0.061 1256 1 1953

2 | 0.055 1276 1 40

3 | 0.055 1276 1 0

Filled

Meta-analysis (exponential form)

| Pooled 95% CI Asymptotic No. of

Method | Est Lower Upper z_value p_value studies

-------+----------------------------------------------------

Fixed | 1.056 1.025 1.089 3.545 0.000 63

Random | 1.119 1.045 1.198 3.234 0.001

Test for heterogeneity: Q= 232.483 on 62 degrees of freedom (p= 0.000)

Moment-based estimate of between studies variance = 0.043


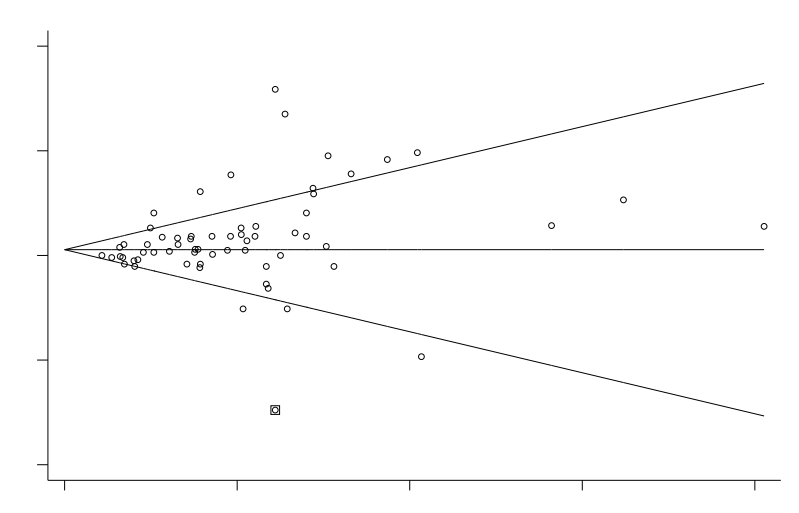


sfigure 4: Funnel plot of PHIP intake and total cancer with trim and fill method

sfigure 5: Sensitivity analyses of PHIP intake and total cancer

sfigure 6: Forest plot of MeIQx intake and total cancer stratified by cancer site

sfigure 7: Forest plot of MeIQx intake and total cancer stratified by geographic location

 sfigure 8: Forest plot of DiMeIQx intake and total cancer stratified by cancer site

sfigure 9: Forest plot of DiMeIQx intake and total cancer stratified by geographic location

sfigure 10: Forest plot of total HCA intake and total cancer stratified by cancer site

Sfigure11: Forest plot of Total HCA intake and total cancer stratified by geographic location

sfigure 12: Forest plot of B(a)P intake and risk of cancer stratified by cancer site

sfigure 13 : Forest plot of B(a)P intake and risk of cancer stratified by geographic location

sfigure 14: Forest plot of PHIP intake and cancer stratified by use CHARRED database

Test(s) of heterogeneity:

Heterogeneity degrees of

statistic freedom P I-squared** Tau-squared

NO CHARRED database144.84 29 0.000 80.0% 0.1438

CHARRED database 41.76 31 0.094 25.8% 0.0035

Overall 192.92 61 0.000 68.4% 0.0332

** I-squared: the variation in ES attributable to heterogeneity)
